# Early Sport Specialization in a Pediatric Population: A Rapid Review of Injury, Function, Performance, and Psychological Outcomes

**DOI:** 10.3390/clinpract15050088

**Published:** 2025-04-29

**Authors:** Emily J. Luo, Jake Reed, John Kyle Mitchell, Emilie Dorrestein, Lulla V. Kiwinda, Steph Hendren, Zoe W. Hinton, Brian C. Lau

**Affiliations:** 1 Department of Orthopaedic Surgery, Duke University School of Medicine, Durham, NC 27710, USA; emilie.dorrestein@duke.edu (E.D.); lulla.kiwinda@duke.edu (L.V.K.); zoe.hinton@duke.edu (Z.W.H.); brian.lau@duke.edu (B.C.L.); 2 UNC Orthopaedics, University of North Carolina School of Medicine, Chapel Hill, NC 27514, USA; jake_reed@med.unc.edu (J.R.); john_mitchell@med.unc.edu (J.K.M.); 3 Duke University Medical Center Library, Durham, NC 27710, USA; stephanie.hendren@duke.edu

**Keywords:** early sport specialization, youth athletes, review, outcomes, sport success, performance, injury, psychological

## Abstract

**Background**: Early sport specialization, involving intensive focus on a single sport for greater than 8 months per year at a prepubescent age, has become increasingly common in young athletes. While early sport specialization is often regarded as essential for success, there is growing concern that the potential risks to young athletes may outweigh the perceived benefits. Although numerous studies have explored early sport specialization, there has been no summative review on the topic in regard to the comprehensive impact on outcomes in these athletes. This study aims to systematically review the literature to determine the impacts of early specialization on injury, function, performance, sport success, and psychological outcomes. **Methods**: A review of Medline (PubMed) was conducted to search for studies relating to early sport specialization in pediatric patients (time of specialization < 18 years old) and outcomes. Extracted information included injury outcomes with a focus on overuse injuries, functional outcomes, performance outcomes, sport success, and psychological outcomes. For studies that reported relevant statistics, *p* < 0.05 was considered statistically significant. All studies were described qualitatively. **Results**: An initial search resulted in a total of 826 studies. After applying inclusion and exclusion criteria, 93 studies were included. A total of 62,327 athletes were included in the study. The average age of study participants was 15.9 years, with an average age at specialization of 11.6 years. Early sport specialization was associated with increased risk of injury, worse functional and physical performance, decreased or no benefit to sport success, and poor psychological outcomes. **Conclusions**: Early sport specialization was associated with increased risk of injury and negative impacts on functional and physical performance measures. There was also no clear advantage regarding sport success. Early sport specialization was linked to harmful effects on athlete psychological well-being. Late specialization, multisport participation, and following training volume guidelines can aid in mitigating these risks.

## 1. Introduction

As the realm of collegiate and professional sports has evolved, there has been an increased emphasis placed on early sport specialization in youth athletics. Early sport specialization is defined as prepubertal children participating in intensive training or competition in organized sports for greater than 8 months per year and excluding participation in other sports to focus on one sport [1]. As athletes commit to college programs at younger ages, many young athletes and parents perceive early sport specialization as crucial for sport success. Furthermore, with the National Collegiate Athletic Association (NCAA) implementing its Name, Image, and Likeness (NIL) policy in June 2021, young athletes may feel even greater pressure to specialize early [2]. Although athletes themselves are often the driving force behind early specialization, parents have also been shown to play a role [3,4]. Additionally, club coaches have been shown to view early sport specialization positively, reinforcing the trend [5]. A popular belief is that an athlete needs at least 10,000 h of practice to be successful in their sport, a misinterpretation that stems from a study conducted by Ericsson et al., which focused largely on musicians and has not been used as a guide in athletic development [6,7]. Even in Ericsson’s study, there was an emphasis made on recovery and avoiding overuse injuries in athletes, which has not been as popularized [6].

Early sport specialization has been linked to numerous problems for these developing athletes. Numerous overuse injuries in the upper and lower extremities have been reported with early sport specialization [8]. Additionally, early sport specialization has been shown to lead to greater rates of reduced sense of accomplishment, sport devaluation, and exhaustion compared to multisport athletes [9]. In contrast, multisport participation has been shown to offer advantages for athletic development and success. For example, 88% of 2018 NFL first-round draft picks played multiple sports in high school [10]. In a survey of over 300 NCAA Division I athletes from 19 different sports, 94.7% had previously played another sport prior to college [11]. In addition to enhancing athletic performance and reducing injury rates, multisport participation, especially unstructured free play, has been linked to improvements in leadership, problem solving skills, decision making, self-control, emotional regulation, and social skills [12].

Current guidelines recommend that adolescent athletes spend no more than 8 months per year in their primary sport, practice no more hours per week than their age (i.e., an 11-year-old should not participate in organized activity for more than 11 h per week) with a cap of 16 h/week [13]. Even with these standards in place, over 80% of parents are unaware of training volume recommendations or the potential negative effects of sport specialization [4].

While numerous studies have explored early sport specialization in general and within certain sports, there has been no summative review on the topic in regard to the comprehensive impact on outcomes in these athletes. Prior reviews have only focused on the risk of injury in regard to early sport specialization [14,15]. Thus, the goal of our study was to systematically review the literature to answer the question on how early sport specialization may impact not only injury, but also functional, performance, sport success, and psychological outcomes.

## 2. Methods

### 2.1. Literature Search and Screening

**Figure 1:**
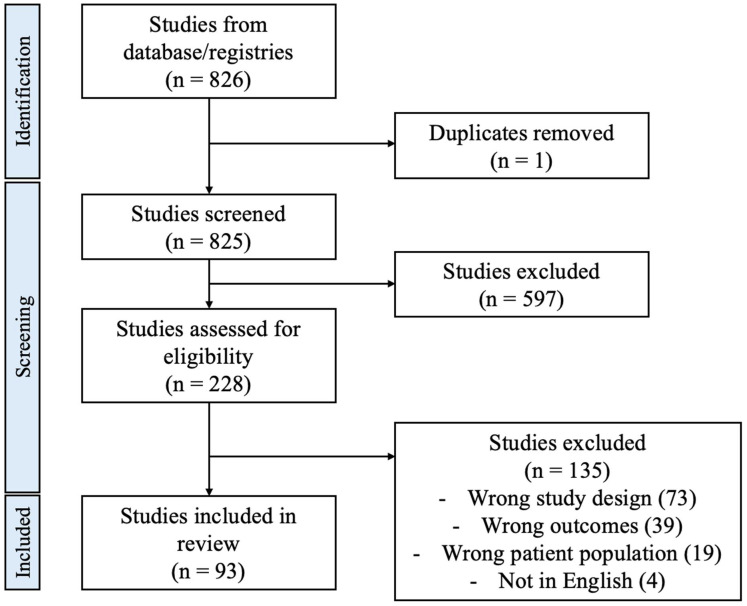
PRISMA flow chart.

This study was a rapid review that followed PRISMA guidelines where applicable for a rapid review article. The search protocol was not registered. We searched Medline (PubMed) using a combination of keywords and database-specific subject headings for the concept of early sport specialization, pediatrics, and outcomes. The complete, reproducible search strategy can be found in Appendix A. The search yielded 826 total citations. All citations were then imported to Covidence (Veritas Health, Melbourne, Australia), a review screening software. One duplicate was removed manually, leaving eight hundred and twenty-five studies to screen. Inclusion criteria involved adolescent athletes/players and/or youth athletes of all sports, discussion of early sport specialization compared to other forms of specialization or non-specialization, and outcomes relating to injury, function, performance, sport success, and psychological impact. Review articles, case reports, non-English texts, opinion pieces, letters to the editor, studies with non-pediatric sport specialization, and studies that did not compare levels of sport specialization were excluded. Following title and abstract screening, 228 full-text studies remained. In full-text screening, 135 studies were excluded for the following reasons: different study design (73 studies), different outcomes (39 studies), different patient population (19 studies), and not in English (4 studies). A total of 93 studies remained for inclusion into our review (Figure 1). All voting disagreements at abstract and full-text levels were resolved with discussion. Manual data extraction was performed with one reviewer extracting each study.

### 2.2. Quality Appraisal and Risk of Bias

All included studies were assessed for risk of bias and study quality using the Methodological Index for Nonrandomized Studies (MINORS) criteria [16]. The MINORS criteria include a 12-item checklist, with each item receiving a score of either 0 (not reported), 1 (inadequately reported), or 2 (adequately reported). Noncomparative and comparative studies have a maximum score of 16 and 24 points, respectively. The full assessment of risk of bias and quality assessment can be found in Appendix B.

### 2.3. Statistical Analysis

Due to the heterogeneity of data, all studies were described qualitatively. Descriptive statistical analysis was performed utilizing R version 3.6.1 (R Foundation, Vienna, Austria).

## 3. Results

### 3.1. Patient Demographics and Cohort

A total of 62,327 athletes were included in the study. The average age of study participants was 15.9 years, with an average age at specialization of 11.6 years. There was a broad spectrum of sport activity described, including youth/school-level, club-level, collegiate-level, professional-level, and world/Olympic-level athletes. The most frequently analyzed sport was soccer, followed by basketball and volleyball. Additional study information including risk of bias assessment scores can be found in Table 1.

### 3.2. Injury Outcomes

Fifty-five studies analyzed injury outcomes associated with level of sport specialization (Table 2). Twenty-four studies found that early sport specialization was significantly associated with injury, a higher risk of total number of injuries (most commonly overuse), and increased time missed from sport. Specific injuries associated with early sport specialization included lower extremity conditions such as patellofemoral pain, patellar tendinopathy, Osgood–Schlatter disease, Cam deformity, and hip and groin dysfunction [24,51,73,76,89]. Upper extremity injuries were primarily overuse injuries of the shoulder and elbow [37,38]. Ha et al. and Okuruwa et al. also noted increased risk of lower back injury, stress fractures, injury history, and concussion in specialized athletes [50,74]. Rate of injury could be modulated by patient-specific factors such as gender, with one study demonstrating that the relationship between injury and sport specialization is more likely in female athletes [26]. Sport specialization may have long-term effects as well, with Wilhelm and colleagues finding that early youth sport specialization was significantly associated with increased injuries in professional baseball [101].

Fifteen studies found no significant difference between sport specialization and injury. Fourteen studies demonstrated mixed results within their analysis. Of these fourteen studies, eight studies found negative outcomes with sport specialization in regard to certain injury outcomes such as overuse injury, pain, or injury necessitating surgery but otherwise noted no significant differences on other injury measures [43,58,63,66,71,94,99,106]. Moseid et al. found negative outcomes associated with sport specialization that were non-significant when adjusting for sex, sport category, and training load at baseline [70]. Four studies found mixed results based on sport specialization [18,46,61,77]. Allahabdi et al. found that while multisport athletes were more likely to suffer a sports injury, single-sport athletes had more medical appointments [18]. Frome and colleagues found that specialized compared to non-specialized athletes were at lower odds of any previous injury, similar odds of previous lower extremity overuse injury, and greater odds of missing more practices [46]. Lenz et al. noted a higher percentage of head and neck injuries in late specialization athletes versus a greater percentage of wrist injury in the early specialization group [61]. Post et al. demonstrated that the impact of sport specialization could be dependent on sport, as overuse injury was prevalent in volleyball but not in basketball or soccer [77].

One study found that early sport specialization was associated with positive outcomes [35]. More specifically, Chen et al. noted that regarding lifetime injury and injury within the last 12 months in rock climbers, late specialization was associated with a significantly higher rate of injury compared to early specialization [35].

### 3.3. Functional and Performance Outcomes

Thirty studies analyzed functional and performance outcomes in regard to sports specialization level (Table 3). Fifteen studies found negative functional and performance outcomes associated with early or high specialization [20,38,40,41,42,43,45,47,53,69,78,80,83,87,98]. More specifically, sport specialization was significantly associated with worse performance testing, particularly on the squat jump, countermovement jump, 20 m sprint, Functional Arm Scale for Throwers (FAST) score, Youth Throwing Score (YTS), and Landing Error Scoring System (LESS) score [20,38,42,53,78,80]. Sport specialization was also noted to be associated with negative biophysical outcomes. Two studies found significant variability on the Drop Vertical Jump task that could be indicative of altered coordination strategies of the hip and knee joints [40,41]. Single-sport athletes also displayed greater asymmetry, lower bone density, decreased quality of life scores, and increased daytime sleepiness [69,83,98]. One study found that even after discontinuing sports, highly specialized youth athletes prior to high school demonstrated clinically significant deficits in lower extremity function as adults [43]. Playing multiple youth sports was also found to be particularly beneficial, with multisport athletes showing improved function, performance, gross motor coordination, higher physical activity levels as adolescents, increased game participation, and longer careers overall [45,47,87].

Nine studies reported no significant differences between sport specialization groups [22,23,33,62,67,79,85,97,100]. Six studies noted mixed findings regarding sport specialization [30,34,52,84,93,96]. Bonnette et al. found that while highly specialized athletes demonstrated greater degrees of coordination compared to the non-specialized group, they were able to break coordinated patterns of joint angle changes, requiring asymmetric demands on the lower extremities [30]. Camp and colleagues found no differences between single- and multisport athletes in regard to range of motion, strength, or pitch velocity, but did note greater external rotation strength in the dominant extremity for multisport athletes when analyzing based on hand dominance [34]. Similarly, Sugimoto found increased ankle plantarflexion but decreased muscular strength of single-sport athletes, but otherwise no significant differences [93]. Two studies noted that the benefits of sport specialization were found to be insignificant depending on the type of functional test or if certain factors such as age were controlled for [52,84]. Interestingly, when sport specialization was further divided into low, moderate or high specialization, moderate specialization was found to have improved movement quality and significantly better LESS scores than either the high or low groups, indicating that a certain amount of specialization could be beneficial [96].

### 3.4. Sport Success

Thirteen studies analyzed sport success outcomes in specialized/early-specialized versus non-specialized/late-specialized athletes (Table 4). Compared to non-specialized/late-specialized athletes, specialized/early-specialized athletes were reported to have mixed results on sport success with three studies showing worse outcomes, four studies showing better outcomes, and six studies showing mixed or non-significant differences in outcomes between the two specialization groups. Ahlquist et al. reported a significant positive correlation between early specialization and the likelihood of being recruited by a college or receiving a college scholarship [17]. However, other studies found that non-specialization was significantly associated with higher level sport participation compared to specialization in one sport and specialization was also not associated with playing at a professional level [31,86]. The majority of the studies showed mixed or similar sport success outcomes between specialized/early-specialized and non-specialized/late-specialized athletes. For example, Meisel et al. showed there was no significant difference between the number of high school athletes that ranked inside the top 250 as compared to outside the top 250 athletes in their class between specialized and non-specialized athletes [68]. Additionally, there was shown to be no significant difference in the percentage of specialized versus non-specialized athletes that received scholarships or had longer college career lengths according to Rugg et al. [88].

### 3.5. Psychological Outcomes

Eighteen studies analyzed psychological outcomes in specialized/early-specialized versus non-specialized/late-specialized athletes (Table 5). Specialized/early-specialized athletes were reported to have either worse or the same psychological outcomes when compared to their non-specialized/late-specialized athlete counterparts, with seven studies showing worse outcomes, nine studies showing similar outcomes, and only two studies showing better outcomes. Chou et al. showed that specialization was significantly associated with higher odds of reporting severe depressive symptoms on PHQ-9 and reduced PedsQL than non-specialized athletes [36]. Other studies also reported that specialization was associated with feelings of excessive competition load and significantly higher fatigue, anxiety, and depressive symptoms than less specialized athletes [92]. In contrast, Zeller et al. reported significantly lower PHQ9 and GAD-7 scores in youth softball athletes who were more specialized compared to those who were less specialized, while HuardPelletier and colleagues elucidated an overall positive correlation between increased specialization and perceptions of sport competency.

## 4. Discussion

### 4.1. Summary

In summary, early sport specialization was generally associated with a higher prevalence of overuse injuries, negative physical and functional metrics, mixed evidence with no definitive advantage in sport success, and potentially worse psychological outcomes (Table 6).

Early sport specialization has been a growing topic of interest. For parents and youth athletes, this desire to play at the elite level has been a major factor in the decision to specialize [107]. However, while information discouraging sport specialization has been widely available, a majority of parents have been found to have no knowledge of sport volume recommendations, and high rates of sport specialization are still being reported [4,78]. This belief has not been shown to be shared by youth sport coaches, the majority of whom recommend playing multiple sports during childhood, thus highlighting the need for providing a more comprehensive overview of the impacts of early sport specialization [108].

#### 4.1.1. Summary of Injury Outcomes

In this study, a total of 54 articles discussed injury-related outcomes relating to sport specialization, with a majority finding that early/high sport specialization was associated with a higher risk of injury. The heightened risk of injuries, particularly overuse injuries, is one of the primary concerns of early or high sport specialization [78,109,110,111,112]. These studies have aided in informing volume recommendations and caps on the number of hours of practice per week for young athletes [13]. In the upper extremity, high levels of early sport specialization have demonstrated increased risk of injury and surgery, particularly in sports with high training volume and repetitive motions such as baseball, swimming, and volleyball [110,111,112,113]. An emphasis has also been placed on adolescent athletes participating in multiple sports or free play, as more unstructured free play can help improve athleticism and increase participation in sports throughout an athlete’s lifetime [12]. Our review of the literature confirms prior reviews that have noted the increased risk of injuries in highly/early-specialized youth athletes [110,111,112].

#### 4.1.2. Summary of Functional/Performance Outcomes and Sport Success

In addition to a heightened risk of injury, 24 studies found that early sport specialization was also either negatively associated with function and physical performance or demonstrated no significant difference with low- or non-specialized athletes. Early sport specialization and performance has been highly studied in sports such as baseball with a high risk of overuse injury and with validated outcome metrics such as the Youth Throwing Score to assess upper extremity health [114]. Biomechanically, the greatest decline in total range of motion in youth baseball players is seen between the ages of 13 and 14, in the year prior to the peak incidence of proximal humeral epiphysiolysis, also known as Little Leaguer’s shoulder. It is thought that this decrease in rotational motion may cause increased stress at the physis during throwing [115]. In regard to sport performance, there have generally been no differences in range of motion, strength, or pitch velocity between multisport and single-sport athletes, thus indicating that the perceived benefits of early sport specialization do not balance the negative impacts on performance and function in the long run [34].

In our study, we also found that across 13 studies describing sport success, there was no clear advantage to early/high sport specialization. Furthermore, in the literature, playing multiple sports has also been shown to benefit sport performance significantly. At the NCAA Division I level, athletes were found to either specialize at an older age or play multiple organized sports prior to college [11,116]. When considering sport success at the professional level, NFL first-round draft picks were more likely to be multisport athletes in high school, and multisport NFL quarterbacks were shown to play in more games, have higher touchdowns per game, more playoff game and Pro Bowl appearances, MVP awards, and Super Bowl victories [10,19]. Similar results were demonstrated with first-round NBA draft picks, with multisport athletes demonstrating significantly greater percentage of total games played, lower likelihood of major career injury, and increased longevity in the NBA [87].

#### 4.1.3. Summary of Psychological Outcomes

In addition to the impacts on physical function and performance, early sport specialization can also have profound effects on the mental and psychological health of these youth athletes. Out of the 18 studies that discussed psychological outcomes relating to sport specialization, the literature was mixed, with seven studies noting a negative association, two finding positive outcomes, and nine studies finding non-significant results. Recent studies have suggested that high sport specialization can be linked to depression, anxiety, burnout, and the internalization of feelings of shame [117]. Early sport specialization has also been associated with a lower health-related quality of life compared to late sport specialization [118]. These findings were similarly reflected in our review. These psychological consequences can be significant, with potentially long-term effects such as jeopardizing return to play, increasing subsequent reinjury risk, and the development of mental health disorders, maladaptive perfectionist traits, clinical eating disorders, or other harmful behaviors that will result in decreased performance, physical health, and overall well-being [117,119]. Thus, it is crucial for athletes, parents, and families to be holistically informed regarding the potential effects of early sport specialization.

### 4.2. Strengths and Limitations

This study had some limitations. Due to the heterogeneity of data and reporting metrics, only qualitative analysis was performed, which could limit the generalizability of our findings. Participant demographics and sports were pooled for many of the studies that were included as well as in our analysis, which may explain the frequency of mixed findings and limit the interpretability of our results. Future sport specialization studies should standardize analysis by patient demographics and compare same or similar sports utilizing well-validated outcome metrics. The strength of this review is the comprehensive synthesis of data regarding sport specialization across multiple outcomes including function, physical performance, and psychological effects, while prior reviews have tended to focus exclusively on injury. We also included a diverse array of sports and levels of competition, which can help inform athletes with a variety of backgrounds and sport involvements.

## 5. Conclusions

In conclusion, early sport specialization was found to increase risk of injury, negatively affect functional, physical performance, and psychological outcomes, as well as limit sport success. Athletes can aim to mitigate these effects through late sport specialization or playing multiple organized sports, increased time of unstructured free play, and adhering to guidelines regarding training volume. Ultimately, we hope that this review can build upon the current body of evidence in the literature to better inform athletes, families, coaches, and providers regarding the potential risks and outcomes associated with early sport specialization.

## Figures and Tables

**Table 1:** Descriptive table. Abbreviations: SD = standard deviation. NR = not reported.

Author Year	Study Design (Retrospective, Prospective, etc.)	Number of Subjects	Age Currently: Mean (SD), Median (SE or Range), or Range	Age at Specialization: Mean (SD), Median (SE or Range), or Range	Sport	Level of Sport Activity	Risk of Bias Assessment
Ahlquist 2020 [17]	Cross-sectional	183	20.1 ± 1.4 (18–23)	Early Sport Specialization: <14 Late Sport Specialization: 14+	Baseball/Softball, Basketball, Cross Country, Football, Golf, Gymnastics, Rowing, Soccer, Swimming/Diving, Tennis, Track and Field, Volleyball, Waterpolo	NCAA Division I	11
Allahabadi 2022 [18]	Survey	203	13.6 ± 3.1	NR	Baseball, Basketball, Football, Soccer	Club, School (level of school not reported)	12
Allahabadi 2023 [19]	Retrospective Cohort	303	NR	NR	Football	NFL	13
Arede 2019 [20]	Cross-Sectional	68	Boys: 12.19 ± 0.58 Girls: 12.03 ± 0.54	Boys: More Specialized: 6.91 ± 1.93, Less Specialized: 10.23 ± 1.36 Girls: More Specialized: 6.50 ± 1.57, Less Specialized: 10.25 ± 1.59	Basketball	U-13	11
Arnold 2019 [21]	Prospective Cohort	159	11.1 ± 1.1	NR	Baseball	Youth League	10
Barfield 2019 [22]	Cross-Sectional	49	12.96 ± 2.32	NR	Baseball, Softball	Youth League	12
Beese 2015 [23]	Cross-Sectional	40	Single Sport Specialized: 15.05 ± 1.2 Multisport Specialized: 15.32 ±1.2	NR	Soccer	Local soccer clubs and Olympic Development Program	12
Bell 2016 [24]	Cross-Sectional	302	15.5 ± 1.2	NR	Basketball, Tennis, Volleyball, Soccer	High School	11
Biese 2020a [25]	Cross-Sectional	1588	15.6 ± 1.1	NR	Volleyball	High School	12
Biese 2020b [26]	Cross-Sectional	2011	13.7 ± 1.6	NR	Baseball, Basketball, Cross Country, Football, Gymnastics, Ice Hockey, Lacrosse, Soccer, Softball, Swimming/Diving, Tennis, Track and Field, Volleyball, Wrestling	High School	10
Biese 2021 [27]	Cross-Sectional	412	20.1 ± 2.0	NR	Baseball, Basketball, Cheer/Dance, Gymnastics, Ice Hockey, Lacrosse, Soccer, Softball, Swimming/Diving, Tennis, Track, Volleyball, Wrestling	Collegiate Club Sports	12
Biese 2022 [28]	Retrospective	466	NR	NR	Basketball, Ice Hockey, Soccer, Softball, Tennis, Track, Volleyball, Wrestling	NCAA Division 1, Club	10
Biese 2024 [29]	Cross-Sectional	178	NR	NR	Archery, Baseball, Basketball, Cross Country, Dance, Equestrian, Football, Golf, Gymnastics, Ice Hockey, Martial Arts, Skiing—Cross Country, Soccer, Swimming/Diving, Tennis, Track and Field, Volleyball, Wrestling	Middle School	10
Bonnette 2023 [30]	Cross-Sectional	49	Non-Specialized: 14.65 ± 1.19 Highly Specialized: 15.00 ± 0.53	NR	Soccer	Middle and High School	13
Bridge 2013 [31]	Retrospective	1006	NR	NR	Boxing, Football, Hockey, Netball, Powerlifting, Rugby, Swimming/Diving	High School	11
Bush 2021 [32]	Cross-Sectional	141	27.9 ± 6.07	Specialization in Youth: 13.4 ± 2.58 (8–16), Specialization in Junior: 17.4 ± 2.21 (8–20)	Weightlifting	USA Weightlifting	13
Butler 2024 [33]	Cross-Sectional	91	12.6 ± 0.9	NR	Baseball, Basketball, Cross Country, Dance, Flag Football, Football, Golf, Gymnastics, Lacrosse, Soccer, Softball, Tennis, Track, Volleyball, Other	High School	11
Camp 2023 [34]	Prospective	115	16.3 ± 1.4	NR	Baseball, Basketball, Football, Golf, Hockey, Roping, Soccer, Swimming/Diving, Wrestling	High School	10
Chen 2022 [35]	Cross-Sectional	101	14 ± 3	11 ± 2 (7–16)	Rock Climbing	National and International level	9
Chou 2023 [36]	Cross-Sectional	2453	Multisport: 15.8 ± 1.2 Specialized: 15.9 ± 1.2	NR	Baseball, Basketball, Cheer, Cross Country, Football, Golf, Ice Hockey, Lacrosse, Soccer, Softball, Swimming/Diving, Tennis, Track, Volleyball, Wrestling	High School	12
Confino 2019 [37]	Cross-Sectional	746	Single Sport: 19.80 Multisport: 20.01	NR	Baseball	MLB	10
Croci 2021 [38]	Cohort	129	Low Specialization: 19.9 ± 1.1 Moderately Specialized: 19.7 ± 1.4 Highly Specialized: 19.9 ± 1.2	13	Baseball	NCAA Division II, NCAA Division III, NAIA, National Club Baseball Association Division	12
Dahab 2019 [39]	Cross-Sectional	97	15.2 ± 1.1	NR	Baseball, Basketball, Cheer, Cross Country, Field Hockey, Football, Golf, Gymnastics, Lacrosse, Rugby, Soccer, Softball, Swimming/Diving, Tennis, Track and Field, Volleyball, Water Polo, Wrestling	High School	12
DiCesare 2019a [40]	Prospective	158	13.4 ± 1.8	NR	Basketball, Soccer, Volleyball	Middle and High School	10
DiCesare 2019b [41]	Prospective	732	13.8 ± 2.0	NR	Basketball, Soccer, Volleyball	Middle School, High School, and College	11
DiStefano 2018 [42]	Cross-Sectional	355	Single Sport: 11 ± 2 Multisport: 11 ± 2	NR	Basketball, Soccer	Elite organizations from suburban/rural areas	12
Dobscha 2023 [43]	Cross-Sectional	356	20 (19–21)	NR	Baseball, Basketball, Cheer, Cross Country, Field Hockey, Football, Gymnastics, Ice Hockey, Lacrosse, Soccer, Softball, Swimming/Diving, Tennis, Track and Field, Volleyball, Wrestling	College students reporting on their high school sports	9
Field 2019 [44]	Cohort	10,138	Male: 12.4 ± 1.6 Female: 12.6 ± 1.6	NR	Baseball, Basketball, Cheer/Gymnastics, Dance, Football, Hockey, Martial Arts, Running, Skating, Soccer, Swimming/Diving, Tennis, Volleyball	NR	10
Fransen 2012 [45]	Cross-Sectional	735	6–12	NR	NR	NR	13
Frome 2019 [46]	Cross-Sectional	2099	13.2 ± 1.8	Age of specialization Total: 9.2 (2.1) Specialized: 9.2 (2.1) Non-Specialized: NR	Soccer	U.S. Soccer Development Academy	10
Gallant 2017 [47]	Cross-Sectional	756	10–11	NR	NR	NR	11
Garcia 2021a [48]	Retrospective	306	15.7 ± 1.1	NR	Baseball/Softball, Basketball, Boxing, Cross Country, CrossFit, Dance, Field Hockey, Gymnastics, Lacrosse, Martial Arts, Nordic Skiing, Soccer, Swimming/Diving, Tennis, Track, Wrestling	Middle and High School	10
Garcia 2021b [49]	Cross-Sectional	258	Male: 15.8 ± 0.9 Female: 15.6 ± 1.4	NR	Cross Country	Middle and High School	10
Ha 2023 [50]	Retrospective	271	NR	NR	Baseball	Korean youth baseball	12
Hall 2015 [51]	Retrospective	546	NR	Single Sport: 14.5 (14.3–14.7) Multisport: 13.8 (13.6–14.0)	Basketball, Soccer, Volleyball	Middle and High School	11
Heath 2021 [52]	Cross-Sectional	147	13.4 ± 2.2	High Specialized: 13.9 (2.2) Low Specialized: 12.1 (1.5)	Basketball, Football, Soccer, Other	NR	11
Herman 2019 [53]	Cross-Sectional	50	23.8 ± 2.5	NR	Basketball, Lacrosse, Soccer, Volleyball	High School Varsity	11
Huard Pelletier 2022 [54]	Cross-Sectional	188	NR	NR	Hockey	Recreation, Competitive, and Elite	11
Huard Pelletier 2024 [55]	Cross-Sectional	971	14.78 ± 1.61	NR	Hockey	Development hockey networks	11
Iona 2022 [56]	Retrospective	169	11.2 ± 2.7	NR	Unspecified Individual: 30 Team: 139	Varies from local to international competition	8
Jayanthi 2015 [57]	Case–Control	1190	13.7 ± 2.3	11.8 (2.6)	NR	NR	12
Jayanthi 2020 [58]	Case–Control	579	14.1 (2.3)	11.62 (2.57)	NR	NR	10
Larson 2019 [59]	Retrospective	81	12–13	8.79 (2.05)	Swimming/Diving	NR	11
Lear 2024 [60]	Cross-sectional Survey	1309	15.1 (1.7)	NR	Softball	NR	10
Lenz 2024 [61]	Cross-sectional survey	133	14.9 (3.8), 15 (8–25)	NR	Diving	Members of USA Diving Association and Collegiate Divers	11
Lima 2020 [62]	Cross-Sectional Survey	321	14.1 (1.7)	10.4 (9.6–11.1)	Basketball	Club	10
Li 2023 [63]	Cross-Sectional Survey	178	13.3 (1.2)	NR	Soccer	Elite (National School Football Winter Camp)	11
McDonald 2019 [64]	Cross-Sectional	143	NR	13.1 (3.82)	Wrestling	NCAA Division I, World/Olympic Team	10
McGowan 2020 [65]	Cross-Sectional Survey	914	12.6 (0.5)	NR	Basketball, Field Hockey, Football, Futsal, Gymnastics, Netball, Rugby, Swimming/Diving, Tennis, Water Polo	National sports competition	10
McGuine 2017 [66]	Prospective	1544	16.1 (1.1)	NR	Baseball, Basketball, Cheer, Dance, Football, Gymnastics, Ice Hockey, Lacrosse, Soccer, Softball, Swimming/Diving, Tennis, Track/Cross Country, Volleyball, Wrestling	High School	11
McKay 2023 [67]	Cross-Sectional	172	22.1 (2.1)	NR	Variety	High School	11
Meisel 2022 [68]	Cross-Sectional Survey	349	16.3 (1.5)	NR	Basketball	Club or High School	10
Miller 2017 [69]	Cross-Sectional Survey	295	NR	NR	Basketball, Soccer, Tennis, Volleyball	High School	11
Moseid 2019 [70]	Cross-Sectional Survey	259	16	NR	Varies	Specialized sport academy high school	11
Murday 2024 [71]	Cross-Sectional	1171	Exclusively specialized: 15.26 Evolved specialized: 15.35 Low-moderately specialized: 15.23	Exclusive: 8.23 Evolved: 9.62	Variety of individual and team	NR	11
Nagano 2023 [72]	Retrospective	1337	Team sports: 30.1 (5.2) Individual: 30.3 (5.1)	NR	Team: Baseball, Basketball, Cheer, Dance, Dance, Handball, Lacrosse, Rhythmic Gymnastics, Soccer, Softball, Volleyball Individual: Archery, Badminton, Ballet, Fencing, Gymnastics, Karate, Kendo, Naginata, Skiing, Soft Tennis, Swimming/Diving, Table Tennis, Tennis, Track and Field	Elementary to High School	11
Nguyen 2023 [73]	Retrospective and Cross-Sectional Questionnaire	66	15–25	10.7 (3.5)	Hockey	Variable	11
Okoruwa 2022 [74]	Cross-Sectional	219	NR	NR	NR	High School	12
Pasulka 2017 [75]	Case–Control	1190	Team Sport: 14.4 (2.2) Individual Sport: 14.1 (2.3)	Team Sports:12.0 (2.7) Individual Sports: 11.2 (2.4)	Variable team and individual	NR	13
Post 2017a [76]	Cross-Sectional	1544	16.1 (1.1)	NR	Variable team and individual	High School	11
Post 2017b [13]	Case–Control	2011	12–18	NR	Baseball, Basketball, Cheer/Dance, Football, Gymnastics, Ice Hockey, Lacrosse, Soccer, Softball, Swimming/Diving, Tennis, Track/Cross Country, Volleyball, Wrestling, Other	Club	12
Post 2020a [77]	Cross-Sectional	716	14.21 (1.5)	NR	Basketball, Soccer, Volleyball	Club Team	10
Post 2020b [78]	Cross-Sectional	551	15.9 ± 1.3	NR	Baseball	Club/High School	12
Post 2021a [79]	Questionnaire	184	9.8	NR	Baseball	Little League	12
Post 2021b [80]	Cross-Sectional	241	9.5 ± 1.6	NR	Baseball	Little League	12
Post 2021c [81]	Cross-Sectional	805	12.9 ± 2.5	Basketball start age: 8.2 ±2.5	Basketball	Travel/Club	12
Post 2024 [82]	Prospective Cohort	130	15.6 (1.3)	NR	Basketball	High School	12
Rauh 2020 [83]	Cross-Sectional	64	15.6 ± 1.4	NR	Cross Country	High School	11
Riehm 2023 [84]	Cross-Sectional	44	Non-Specialized: 14.68 (1.2) Specialized: 15.04 (0.53)	NR	Soccer	Club	11
Root 2019 [85]	Retrospective	131	10.9 ± 2.9 Low: 7.95 ± 2.64 Moderate: 11.67 ± 2.78 High: 10.87 ± 2.63	NR	Gymnastics	Club	13
Ross 2022 [86]	Retrospective	101	>18	Overall: 13 ± 4 Professional: 14 ± 3 Collegiate: 13 ± 4 Junior: 11 ± 4	Ice Hockey	Professional, collegiate, junior	10
Rugg 2018 [87]	Retrospective	237	Multisport: 21.0 ± 1.4 Single Sport: 20.8 ± 1.3	NR	Basketball	Professional	11
Rugg 2021 [88]	Retrospective Cohort	1550	NR	<15 years	Baseball, Basketball, Cross Country, Fencing, Field Hockey, Football, Golf, Gymnastics, Ice Hockey, Lacrosse, Rowing, Rugby, Skiing, Soccer, Softball, Swimming/Diving, Tennis, Track and Field, Volleyball, Water Polo, Wrestling	NCAA	11
Sheppard 2020 [89]	Retrospective Cohort	187	20.8 ± 1.9	14.8 ± 3.6	Ice hockey	NCAA Division III	11
Söderström 2023 [90]	Retrospective	1026	21	NR	Soccer	Club	9
Staub 2020 [91]	Cohort study	1705	18	11	Swimming	Club	14
Steinl 2021 [10]	Cross-sectional	318	Specialized: 22.0 ± 1.0 Multisport: 22.3 ± 1.1	NR	Football Multisport athletes: Baseball, Basketball, Golf, Hockey, Lacrosse, Rugby, Soccer, Tennis, Track, Wrestling	Professional	13
Stockbower 2022 [92]	Cross-sectional	186	15.3 ± 1.3	NR	NR	High School	13
Sugimoto 2019 [93]	Cross-Sectional	236	12–18	NR	30 different sports	Club/High School	13
Sweeney 2021 [94]	Retrospective survey	473	32.4 (26.3, 41.2)	Early: 8 (5, 10) Late: 16 (14, 18)	Gymnastics	College	11
Swindell 2019 [11]	Cross-Sectional	303	19.9 ± 1.52	Overall: 14.9 ± 3.06	Archery, Baseball, Basketball, Cross Country, Fencing, Field Hockey, Football, Golf, Lacrosse, Rowing, Soccer, Softball, Squash, Swimming/Diving, Tennis, Track and Field, Volleyball, Water polo, Wrestling	NCAA Division 1	11
Valenzuela-Moss 2024 [95]	Prospective	126	Grades 7–11	NR	NR	Middle School/High School	14
Venrick 2021 [96]	Cross-sectional and Prospective Cohort	1950	17–23	NR	NR	Varsity, High School	13
Watson 2019 [97]	Prospective Cohort	52	12.9–17.9	NR	Soccer	Club	13
Watson 2022 [98]	Cross-Sectional	1482	14–18	NR	Volleyball	High School	13
Whatman 2023 [99]	Cross-Sectional	1504	14–19	<12 years old	Top 10: Alpine Skiing, Badminton, Basketball, Dance, Football, Ice Hockey, Running, Soccer, Swimming/Diving, Volleyball	High School	13
Whatman 2021 [100]	Cross-Sectional	238	11–16	NR	Badminton, Basketball, Basketball, Cheer, Dance, Figure Skating, Football, Gymnastics, Hockey, Horseback Riding, Karate, Lacrosse, Martial Arts, Mixed Martial Arts, Parkour, Ringette, Rock Climbing, Skateboarding, Soccer, Sprinting, Swimming, Taekwondo, Tennis, Volleyball	Junior High	13
Wilhelm 2017 [101]	Descriptive epidemiological	102	22–40	8.91 (3.7)	Baseball	Professional, survey about youth career	11
Wilkins 2023 [102]	Cross-Sectional	752	19.9 ± 1.5	15.6	Baseball	College	11
Wilkins 2024 [103]	Qualitative study, Online Teleconference	25	Undergraduate college	NR	Baseball	College	9
Xiao 2021 [104]	Cross-Sectional Survey	1081	Youth: 14.7 ± 1.6 College/Pro: 19.7 ± 1.7	Youth: 339 (44.2%) College/Professional: 65 (25.9%)	Soccer	Youth	11
Zeller 2024 [105]	Cross-Sectional Survey	1283	15.1 ± 1.7	NR	Softball	Youth	13
Zoellner 2022 [106]	Cross-Sectional Survey	414	12.8 ± 1.1	NR	Soccer	Youth	13

**Table 2:** Injury outcomes.

Author Year	Sport	Level of Sport Activity	Injury Outcomes	Early Sport Specialization Bad/Good/Same/Mixed
Ahlquist 2020 [17]	Baseball/Softball, Basketball, Cross Country, Football, Golf, Gymnastics, Rowing, Soccer, Swimming/Diving, Tennis, Track and Field, Volleyball, Waterpolo	NCAA Division I	Early sport specialization was significantly associated with a greater total number of injuries and increased time missed for injury compared to late specialization.	Bad
Allahabadi 2022 [18]	Baseball, Basketball, Football, Soccer	Club, School (level of school not reported)	Multisport athletes were more likely to present to clinic for a sports-related injury compared to single-sport athletes, but single-sport athletes had a statistically significantly greater number of medical appointments for sport injuries.	Mixed
Arnold 2019 [21]	Baseball	Youth League	Specialization in baseball amongst youth athletes was associated with higher frequency of injury.	Bad
Bell 2016 [24]	Basketball, Tennis, Volleyball, Soccer	High School	Overuse knee injuries were associated with high sport specialization.	Bad
Biese 2020a [25]	Volleyball	High School	Moderate and high sport specialization were associated with statistically significantly increased odds of musculoskeletal injury in past 12 months.	Bad
Biese 2020b [26]	Baseball, Basketball, Cross Country, Football, Gymnastics, Ice Hockey, Lacrosse, Soccer, Softball, Swimming/Diving, Tennis, Track and Field, Volleyball, Wrestling	High School	Highly specialized athletes were more likely to report acute and overuse injuries, but this relationship was dependent on sex (more likely in female athletes).	Bad
Biese 2021 [27]	Baseball, Basketball, Cheer/Dance, Gymnastics, Ice Hockey, Lacrosse, Soccer, Softball, Swimming/Diving, Tennis, Track, Volleyball, Wrestling	Collegiate Club Sports	No differences between high school sport specialization and college club sport injuries.	Same
Bush 2021 [32]	Weightlifting	USA Weightlifting	Before age 21, specializing in weightlifting at the youth level was significant for increased occurrence of injury.	Bad
Camp 2023 [34]	Baseball, Basketball, Football, Golf, Hockey, Roping, Soccer, Swimming/Diving, Wrestling	High School	No statistically significant difference in history of injury between multisport and single sport athletes.	Same
Chen 2022 [35]	Rock Climbing	National and International level	For lifetime injury, late sport specialization was associated with a significantly higher rate compared to early specialization. For injury within the last 12 months, late specialization was associated with a significantly higher rate compared to early and non-specialized athletes.	Good
Confino 2019 [37]	Baseball	MLB	Single-sport athletes had significantly higher number of upper extremity injuries and recurrent elbow injuries compared to multisport athletes.	Bad
Croci 2021 [38]	Baseball	NCAA Division II, NCAA Division III, NAIA, National Club Baseball Association Division	High sport specialization more likely to report shoulder and elbow injuries.	Bad
Dahab 2019 [39]	Baseball, Basketball, Cheer, Cross Country, Field Hockey, Football, Golf, Gymnastics, Lacrosse, Rugby, Soccer, Softball, Swimming/Diving, Tennis, Track and Field, Volleyball, Water Polo, Wrestling	High School	No differences were evident in injury history among the specialization groups.	Same
Dobsacha 2023 [43]	Baseball, Basketball, Cheer, Cross Country, Field Hockey, Football, Gymnastics, Ice Hockey, Lacrosse, Soccer, Softball, Swimming/Diving, Tennis, Track and Field, Volleyball, Wrestling	College students reporting on their high school sports	High-specialization athletes reported significantly greater foot/ankle pain and sport-related knee injury compared to low-specialization group, otherwise there were no statistically significant differences.	Mixed
Field 2019 [44]	Baseball, Basketball, Cheer/Gymnastics, Dance, Football, Hockey, Martial Arts, Running, Skating, Soccer, Swimming/Diving, Tennis, Volleyball	NR	Risk of injury with sports specialization was associated with female gender and varied by sport.	Mixed
Frome 2019 [46]	Soccer	U.S. Soccer Development Academy	Specialized athletes had lower odds of any previous injury, similar odds of previous lower extremity overuse injury, and greater odds of missing more practices due to injury.	Mixed
Garcia 2021a [48]	Baseball/Softball, Basketball, Boxing, Cross Country, CrossFit, Dance, Field Hockey, Gymnastics, Lacrosse, Martial Arts, Nordic Skiing, Soccer, Swimming/Diving, Tennis, Track, Wrestling	Middle and High School	No statistically significant difference in frequency of sport specialization based on injury status.	Same
Garcia 2021b [49]	Cross Country	Middle and High School	No statistically significant differences between sport specialization levels for running-related injuries.	Same
Ha 2023 [50]	Baseball	Korean youth baseball	Excessive practice and a lack of rest during middle school (growth spurt period) can significantly increase the risk of lower back problems in young baseball players.	Bad
Hall 2015 [51]	Basketball, Soccer, Volleyball	Middle and High School	Single-sport athletes were at increased risk of patellofemoral pain, patellar tendinopathy, and Osgood–Schlatter Disease compared to multiple sport athletes.	Bad
Iona 2022 [56]	Unspecified Individual: 30 Team: 139	Varies from local to international competition	There were no statistically significant differences between sport specialization groups in regard to rest from sports for overuse injuries.	Same
Jayanthi 2015 [57]	NR	NR	Sport specialization was an independent risk factor for injury.	Bad
Jayanthi 2020 [58]	NR	NR	Moderate- and high-specialization athletes were at increased odds for overuse injury. No statistically significant differences in serious overuse injuries or reinjury.	Mixed
Lenz 2024 [61]	Diving	Members of USA Diving Association and Collegiate Divers	Late sport specialization had significantly higher percentage of head/neck injuries while early sport specialization had significantly higher percentage of wrist injuries, otherwise there were no significant differences.	Mixed
Li 2023 [63]	Soccer	Elite (National School Football Winter Camp)	Early sport specialization had a significantly higher odds of any injury, otherwise there were no significant differences.	Mixed
McDonald 2019 [64]	Wrestling	NCAA Division I, World/Olympic Team	Early sport specialization was associated with significantly greater number of major injuries prior to college.	Bad
McGowan 2020 [65]	Basketball, Field Hockey, Football, Futsal, Gymnastics, Netball, Rugby, Swimming/Diving, Tennis, Water Polo	National sports competition	No significant differences in injury between specialization groups	Same
McGuine 2017 [66]	Baseball, Basketball, Cheer, Dance, Football, Gymnastics, Ice Hockey, Lacrosse, Soccer, Softball, Swimming/Diving, Tennis, Track/Cross Country, Volleyball, Wrestling	High School	Moderate- and high-specialization athletes were at significantly increased odds for any lower extremity injury and chronic lower injury. There were no significant differences in acute lower injury or injury requiring surgery.	Mixed
McKay 2023 [67]	Variety	High School	High-specialization group had significantly greater number of previous injuries reported.	Same
Meisel 2022 [68]	Basketball	Club or High School	No significant relationship between early specialization prior to age 14 years and basketball-related injury.	Same
Moseid 2019 [70]	Varies	Specialized sport academy high school	Early sport specialization was associated with an increased risk of acute injuries, but this association was modified by sex, sport category, and training load at baseline, and no longer significant after adjustment for these factors.	Mixed
Murday 2024 [71]	Variety of individual and team	NR	For acute and overuse injuries, the exclusive highly specialized group did not differ from the evolved highly specialized group. However, the exclusive highly specialized group differed from the low–moderately specialized group, and the evolved highly specialized group differed from the low–moderately specialized group. For overuse and serious overuse injuries, there was no difference between the exclusive highly specialized and evolved highly specialized groups, the exclusive highly specialized and low–moderately specialized groups, and the evolved highly specialized and low–moderately specialized groups.	Mixed
Nagano 2023 [72]	Team: Baseball, Basketball, Cheer, Dance, Dance, Handball, Lacrosse, Rhythmic Gymnastics, Soccer, Softball, Volleyball Individual: Archery, Badminton, Ballet, Fencing, Gymnastics, Karate, Kendo, Naginata, Skiing, Soft Tennis, Swimming/Diving, Table Tennis, Tennis, Track and Field	Elementary to High School	Sport specialization was significantly associated with a greater prevalence of overuse injuries.	Bad
Nguyen 2023 [73]	Hockey	Variable	Moderate and high specialization were significantly associated with increased odds of Cam deformity.	Bad
Okoruwa 2022 [74]	NR	High School	Moderate and high specialization were significantly associated with stress fractures, injury history, and history of concussion.	Bad
Pasulka 2017 [75]	Variable team and individual	NR	Single-sport-specialized athletes in individual sports accounted for a higher proportion of overuse injuries and serious overuse injuries, but a lower proportion of acute injuries compared to single-sport-specialized athletes involved in team sports.	Bad
Post 2017a [76]	Variable team and individual	High School	Moderate- and high-specialization athletes had significantly increased odds of previous lower extremity injury.	Bad
Post 2017b [13]	Baseball, Basketball, Cheer/Dance, Football, Gymnastics, Ice Hockey, Lacrosse, Soccer, Softball, Swimming/Diving, Tennis, Track/Cross Country, Volleyball, Wrestling, Other	Club	High-specialization athletes had significantly greater injuries and overuse injuries.	Bad
Post 2020a [78]	Basketball, Soccer, Volleyball	Club Team	Volleyball was associated with significant association between high sport specialization and overuse injury. Basketball and soccer did not demonstrate significant differences between sport specialization and overuse injury.	Mixed
Post 2024 [82]	Basketball	High School	There was no difference in injury risk between highly specialized and low-specialized athletes. Injury risk may be specific to certain behaviors such as year-round play and participation in skills camps.	Same
Post 2021c [81]	Basketball	Travel/Club	High-specialization athletes had significantly greater number of injuries	Bad
Ross 2022 [86]	Ice Hockey	Professional, collegiate, junior	No significant difference between groups and total injuries.	Same
Rugg 2018 [87]	Basketball	Professional	Single-sport-specialized athletes had a significantly higher proportion sustaining major injury.	Bad
Rugg 2021 [88]	Baseball, Basketball, Cross Country, Fencing, Field Hockey, Football, Golf, Gymnastics, Ice Hockey, Lacrosse, Rowing, Rugby, Skiing, Soccer, Softball, Swimming/Diving, Tennis, Track and Field, Volleyball, Water Polo, Wrestling	NCAA	No significant difference between groups in sustaining collegiate injury.	Same
Sheppard 2020 [89]	Ice hockey	NCAA Division III	Early ice hockey specialization may be detrimental to hip and groin function in collegiate ice hockey athletes.	Bad
Steinl 2021 [10]	Football Multisport athletes: Baseball, Basketball, Golf, Hockey, Lacrosse, Rugby, Soccer, Tennis, Track, Wrestling	Professional	No significant difference in missed games due to upper extremity injuries.	Same
Stockbower 2022 [92]	NR	High School	No significant difference in history of time-loss injury between groups.	Same
Sweeney 2021 [94]	Gymnastics	College	Early specialization was significantly associated with sustaining in injury that resulted in surgery. Otherwise, there were no significant differences in college time-loss injury, retirement during college, or stress fracture during college.	Mixed
Watson 2022 [98]	Volleyball	High School	High specialization was significantly associated with increased rate of injury.	Bad
Whatman 2023 [99]	Top 10: Alpine Skiing, Badminton, Basketball, Dance, Football, Ice Hockey, Running, Soccer, Swimming/Diving, Volleyball	High School	High specialization was significantly associated with all musculoskeletal injuries. There were no significant differences in lower extremity musculoskeletal injuries and concussions.	Mixed
Whatman 2021 [100]	Badminton, Basketball, Basketball, Cheer, Dance, Figure Skating, Football, Gymnastics, Hockey, Horseback Riding, Karate, Lacrosse, Martial Arts, Mixed Martial Arts, Parkour, Ringette, Rock Climbing, Skateboarding, Soccer, Sprinting, Swimming, Taekwondo, Tennis, Volleyball	Junior High	No significant association between specialization and injury history.	Same
Wilkins 2023 [102]	Baseball	College	Early baseball specialization as a youth baseball player may not impact throwing arm health in college baseball athletes.	Same
Wilhelm 2017 [101]	Baseball	Professional, survey about youth career	Early sport specialization associated with significantly increased injuries as a professional.	Bad
Xiao 2021 [104]	Soccer	Youth	High specialization in female youth soccer players is associated with an increased likelihood of sustaining a serious injury.	Bad
Zoellner 2022 [106]	Soccer	Youth	High sport specialization associated with significantly increased odds of gradual onset injury on both unadjusted and adjusted analysis. Otherwise, there were no statistically significant differences.	Mixed

**Table 3:** Functional and performance outcomes. Abbreviations: LESS = Landing Error Scoring System. TGMD2 = Test of Gross Motor Development. FAST = Functional Arm Scale for Throwers.

Author Year	Sport	Level of Sport Activity	Functional Outcomes	Early Sport Specialization Bad/Good/Same/Mixed
Arede 2019 [20]	Basketball	U-13	Sport specialization was significantly negatively associated with performance testing.	Bad
Barfield 2019 [22]	Baseball, Softball	Youth League	No significant different between specialization groups.	Same
Beese 2015 [23]	Soccer	Local soccer clubs and Olympic Development Program	No difference between groups on LESS testing.	Same
Bonnette 2023 [30]	Soccer	Middle and High School	The results indicate that the highly specialized athletes tended to exhibit greater degrees of coordination but also the ability to break the coordinated patterns of joint angle changes to execute a cutting maneuver, which requires asymmetric demands on the lower extremities while planting on one leg and changing direction.	Mixed
Butler 2024 [33]	Baseball, Basketball, Cross Country, Dance, Flag Football, Football, Golf, Gymnastics, Lacrosse, Soccer, Softball, Tennis, Track, Volleyball, Other	High School	There was no difference in proficiency on Test of Gross Motor Development between specialization levels.	Same
Camp 2023 [34]	Baseball, Basketball, Football, Golf, Hockey, Roping, Soccer, Swimming/Diving, Wrestling	High School	No differences between groups on range of motion, strength, or pitch velocity. External rotation strength in dominant extremity was significantly greater in multisport athletes than single sport.	Mixed
Croci 2021 [38]	Baseball	NCAA Division II, NCAA Division III, NAIA, National Club Baseball Association Division	High specialization was significantly associated with poorer throwing arm function.	Bad
DiCesare 2019a [40]	Basketball, Soccer, Volleyball	Middle and High School	Sport specialization was significantly associated with biomechanical changes that are indicative of potentially compromised neuromuscular control that may increase injury risk pre- to post-puberty in sport-specialized female athletes.	Bad
DiCesare 2019b [41]	Basketball, Soccer, Volleyball	Middle School, High School, and College	Sport specialization was associated with increased variability of critical hip- and knee-joint couplings responsible for effective landing during the DVJ. Altered coordination strategies that involve the hip and knee joints may underlie unstable landings, inefficient force-absorption strategies, or greater contact forces that can place the lower extremities at risk for injury (or a combination of these).	Bad
DiStefano 2018 [42]	Basketball, Soccer	Elite organizations from suburban/rural areas	Sport specialization was associated with worse neuromuscular control.	Bad
Dobsacha 2023 [43]	Baseball, Basketball, Cheer, Cross Country, Field Hockey, Football, Gymnastics, Ice Hockey, Lacrosse, Soccer, Softball, Swimming/Diving, Tennis, Track and Field, Volleyball, Wrestling	College students reporting on their high school sports	Even after discontinuing sports, young adults who were highly specialized in youth sports before high school reported clinically important deficits in lower extremity function.	Bad
Fransen 2012 [45]	NR	NR	Playing multiple sports was significantly associated with improved function, performance, and gross motor coordination.	Bad
Gallant 2017 [47]	NR	NR	Sport sampling should be promoted in childhood because it may be linked to higher physical levels during adolescence.	Bad
Heath 2021 [52]	Basketball, Football, Soccer, Other	NR	High sport specialization was associated with better movement quality; however, this relationship was not significant when controlling for age.	Mixed
Herman 2019 [53]	Basketball, Lacrosse, Soccer, Volleyball	High School Varsity	LESS scores were lower in athletes who had a history of multisport high school varsity participation compared with those who had a history of single-sport or no participation in these sports at this level. Multisport high school varsity participation in these sports may result in improved neuromuscular performance and potentially reduced injury risks as adults.	Bad
Lima 2020 [62]	Basketball	Club	No difference between specialization groups on functional outcomes.	Same
McKay 2023 [67]	Variety	High School	No difference between specialization groups on functional outcomes.	Same
Miller 2017 [69]	Basketball, Soccer, Tennis, Volleyball	High School	Clinicians should be aware that single-sport male athletes may display deficits in dynamic balance, potentially increasing their risk of injury.	Bad
Post 2020b [78]	Baseball	Club/High School	High specialization in baseball, particularly for pitchers, was associated with upper extremity overuse injury history and worse throwing-arm health in high school baseball athletes.	Bad
Post 2021a [79]	Baseball	Little League	Sport specialization was not significantly associated with youth throwing scores.	Same
Post 2021b [80]	Baseball	Little League	Sport specialization was negatively associated with throwing score.	Bad
Rauh 2020 [83]	Cross Country	High School	A high level of sport specialization in high school female distance runners may be associated with a heightened risk for low bone mineral density.	Bad
Riehm 2023 [84]	Soccer	Club	Center of gravity trajectories of specialized and non-specialized athletes differed with respect to movement variability.	Mixed
Root 2019 [85]	Gymnastics	Club	There were generally no differences between groups for gymnastics fitness tasks.	Same
Rugg 2018 [87]	Basketball	Professional	Multisport athletes in high school participated in more games and had longer careers than those who participated in a single sport.	Bad
Sugimoto 2019 [93]	30 different sports	Club/High School	There were significant differences between single- and multisport athletes in regard to ankle plantarflexion range of motion and knee extension muscular strength. Otherwise, there was no significant differences between groups.	Mixed
Venrick 2021 [96]	NR	Varsity, High School	Women reporting moderate sport specialization had improved movement quality and significantly better LESS scores compared to those with high/low specialization.	Mixed
Watson 2019 [97]	Soccer	Club	No differences between specialization groups in regard to VO2 Max or time to exhaustion	Same
Watson 2022 [98]	Volleyball	High School	Highly specialized female volleyball athletes demonstrate decreased quality of life and increased daytime sleepiness.	Bad
Whatman 2021 [100]	Badminton, Basketball, Basketball, Cheer, Dance, Figure Skating, Football, Gymnastics, Hockey, Horseback Riding, Karate, Lacrosse, Martial Arts, Mixed Martial Arts, Parkour, Ringette, Rock Climbing, Skateboarding, Soccer, Sprinting, Swimming, Taekwondo, Tennis, Volleyball	Junior High	Level of sport specialization was not associated with a range of physical performance measures.	Same

**Table 4:** Sport success outcomes.

Author Year	Sport	Level of Sport Activity	Sport Success Outcomes	Early Sport Specialization Bad/Good/Same/Mixed
Ahlquist 2020 [17]	Baseball/Softball, Basketball, Cross Country, Football, Golf, Gymnastics, Rowing, Soccer, Swimming/Diving, Tennis, Track and Field, Volleyball, Waterpolo	NCAA Division I	Compared to late specialization, early sport specialization is associated with increased odds of being recruited, receiving scholarship, and receiving a full scholarship.	Good
Allahabadi 2023 [19]	Football	NFL	In the regular season, non-specialized (multisport) NFL QBs have significantly more games played per season, touchdowns thrown per game, pass yards per game, higher QB rating compared to specialized (single-sport) QBs. There were no significant differences in passing completion, interceptions, and rush yards per game compared to specialized QBs. In the playoffs, non-specialized (multisport) QBs have significantly more games played per season than specialized (single-sport) QBs. There were no significant differences in touchdowns per game, pass completion, pass yards per game, interceptions per game, rush yards per game, and QB rating compared to specialized QBs. Non-specialized QBs have significantly higher rates of Pro Bowl appearances, MVP awards, and Super Bowl victories per athlete compared to non-specialized QBs.	Bad
Bridge 2013 [31]	Boxing, Football, Hockey, Netball, Powerlifting, Rugby, Swimming/Diving	High School	Non-specialization was significantly associated with higher level sport participation compared to specialization in one sport.	Bad
HuardPelletier 2022 [54]	Hockey	Recreation, Competitive, and Elite	There was a significant difference in magnitude (*p* < 0.001) that exists in sport specialization levels based on the playing level of the ice hockey players. Specifically, there are fewer competitive players in the high specialization group and more in the low specialization group than at the other two playing levels. Elites also have a significantly larger proportion of highly specialized athletes and a lower proportion of athletes with low specialization.	Good
Meisel 2022 [68]	Basketball	Club or High School	There is no significant difference between the number of athletes that ranked inside the top 250 as compared to outside the top 250 athletes in their class comparing specialization vs. non-specialization.	Same
Ross 2022 [86]	Ice hockey	Professional, collegiate, junior	Specializing exclusively in ice hockey earlier in life was not associated with playing professionally.	Bad
Rugg 2021 [88]	Baseball, Basketball, Cross Country, Fencing, Field Hockey, Football, Golf, Gymnastics, Ice Hockey, Lacrosse, Rowing, Rugby, Skiing, Soccer, Softball, Swimming/Diving, Tennis, Track and Field, Volleyball, Water Polo, Wrestling	NCAA	There is no significant difference in the percentage of specialized vs. non-specialized athletes that received scholarships or had longer college career lengths.	Same
Söderström 2023 [90]	Soccer	Club	There was no significant association between early specialization and elite adult sports participation.	Same
Staub 2020 [91]	Swimming	Club	Less specialization was associated with greater success by having a higher FINA point score at age 18 especially when comparing national team members with non-national team members. There was an association in earlier specialization and entry into the top 100 with higher FINA point scores at age 18. Being listed in more than one stroke correlated with success at age 18.	Mixed
Steinl 2021 [10]	Football Multisport athletes: Baseball, Basketball, Golf, Hockey, Lacrosse, Rugby, Soccer, Tennis, Track, Wrestling	Professional	There is no significant difference in specialized and non-specialized NFL players making more than one Pro Bowl and being selected for the Pro Bowl. There is no significant difference in specialized and non-specialized NFL players that are currently active players.	Same
Watson 2019 [97]	Soccer	Club	No significant difference between the number of years playing soccer between specialized and non-specialized female athletes.	Same
Xiao 2021 [104]	Soccer	Youth	Specialization in soccer at a young age was reported by youth participants as a way they felt helped advance them to a higher level in their athletic career; however, this choice often led to feelings of burnout and added pressure.	Good

**Table 5:** Psychological outcomes.

Author Year	Sport	Level of Sport Activity	Psychologic Outcomes	Early Sport Specialization Bad/Good/Same/Mixed
Biese 2024 [29]	Archery, Baseball, Basketball, Cross Country, Dance, Equestrian, Football, Golf, Gymnastics, Ice Hockey, Martial Arts, Skiing—Cross Country, Soccer, Swimming/Diving, Tennis, Track and Field, Volleyball, Wrestling	Middle School	No significant difference in the average identified regulation score between single- and multisport athletes.	Same
Chou 2023 [36]	Baseball, Basketball, Cheer, Cross Country, Football, Golf, Ice Hockey, Lacrosse, Soccer, Softball, Swimming/Diving, Tennis, Track, Volleyball, Wrestling	High School	Specialization was significantly associated with higher odds of reporting severe depressive symptoms on PHQ-9 and reduced PedsQL than non-specialization.	Bad
Dahab 2019 [39]	Baseball, Basketball, Cheer, Cross Country, Field Hockey, Football, Golf, Gymnastics, Lacrosse, Rugby, Soccer, Softball, Swimming/Diving, Tennis, Track and Field, Volleyball, Water Polo, Wrestling	High School	No significant differences between quality of life or depression in specialized vs. non-specialized athletes.	Same
Garcia 2021b [49]	Cross Country	Middle and High School	No significant difference between specialized vs. non-specialized athletes in reporting quality of life, sleep quality, or sleep duration in male and female middle and high school runners.	Same
HuardPelletier 2022 [54]	Hockey	Recreation, Competitive, and Elite	There is no difference in early specialization vs. non-specialization when it comes to athletes realizing their sport competencies.	Same
HuardPelletier 2024 [55]	Hockey	Development hockey networks	More specialized athletes tend to have stronger perceptions of their competencies.	Good
Lima 2020 [62]	Basketball	Club	Enjoyment, motivation for deliberate practice, and motivation for achievement and competition do not appear to be negatively influenced by early specialization.	Same
McDonald 2019 [64]	Wrestling	NCAA Division I, World/Olympic Team	No significant difference between the percentage of early- and late-specialization athletes in feeling as if they needed to specialized in order to be an elite wrestler.	Same
Okoruwa 2022 [74]	NR	High School	Three-point specialization, six-point specialization, and self-reported specialization showed no significant difference between athletes that were labeled as highly, moderate-, or low-specialized and were worried about their weight.	Same
Post 2017b [13]	Baseball, Basketball, Cheer/Dance, Football, Gymnastics, Ice Hockey, Lacrosse, Soccer, Softball, Swimming/Diving, Tennis, Track/Cross Country, Volleyball, Wrestling, Other	Club	There was a significantly higher percentage of athletes that reported missing time with friends that were highly specialized compared to moderately and low-specialized athletes.	Bad
Stockbower 2022 [92]	NR	High School	There was significantly higher fatigue, anxiety, and depressive symptoms reported for higher specialized athletes than moderately specialized athletes.	Bad
Swindell 2019 [11]	Archery, Baseball, Basketball, Cross Country, Fencing, Field Hockey, Football, Golf, Lacrosse, Rowing, Soccer, Softball, Squash, Swimming/Diving, Tennis, Track and Field, Volleyball, Water polo, Wrestling	NCAA Division 1	Among specialized athletes, there was a significant difference in those wanting to specialize in an individual sport vs. a team sport for the reasons of lacking time for multiple sports, wanting to acquire a college scholarship, and wanting to go professional.	Same
Valenzuela-Moss 2024 [95]	NR	Middle School/High School	There was no significant increase in burnout among specialized athletes as age increased, but there was significant school-related burnout among specialized athletes as they moved from middle to high school.	Bad
Watson 2019 [97]	Soccer	Club	There were significantly lower (worse) scores for fatigue, soreness, mood, and sleep quality in specialized athletes vs. non-specialized athletes.	Bad
Watson 2022 [98]	Volleyball	High School	No significant difference in emotional, social, school, and psychosocial function between specialized and non-specialized athletes.	Same
Wilkins 2024 [103]	Baseball	College	Moderate- and high-level specialists described higher levels of passion, participating in deliberate practice, higher levels of performance-related stress, and a desire to quit sports more than low-level specialists.	Bad
Zeller 2024 [105]	Softball	Youth	There were significantly lower PHQ9 and GAD-7 scores in athletes that were more specialized compared to those who were less specialized.	Good

**Table 6:** Summary table of study breakdown and outcomes.

Outcome Category	Number of Studies	Study Breakdown Relative to Early/High Sport Specialization	Conclusion
Injury	54	- Significant negative association: 24 studies (44.4%) - Mixed results: 14 studies (25.9%) - Significantly improved injury outcomes: 1 study (1.9%) - Non-significant: 15 studies (27.8%)	Early/High sport specialization was generally associated with higher risk of injury.
Function/Performance	30	- Significant negative association: 15 studies (50.0%) - Mixed results: 6 studies (20.0%) - Non-significant: 9 studies (30.0%)	Early/High sport specialization was generally associated with worser function and performance outcomes.
Sport Success	13	- Significant negative association: 3 studies (23.1%) - Significantly improved sport success: 4 studies (30.8%) - Mixed/Non-significant: 6 studies (46.2%)	Early/High sport specialization demonstrated mixed evidence but no clear advantage in regard to sport success.
Psychological	18	- Significant negative association: 7 studies (38.9%) - Significantly improved psychological outcomes: 2 studies (11.1%) - Non-significant: 9 studies (50.0%)	Early/High sport specialization may impact psychological outcomes.

## Data Availability

The original data presented in the study are openly available.

## Appendix B. Full Quality Assessment and Risk of Bias

**Table:**

MINORS	A Clearly Stated Aim	Inclusion of Consecutive Patients	Prospective Collection of Data	Endpoints Appropriate to the Aim of the Study	Unbiased Assessment of Study Endpoint	Follow-Up Period Appropriate to the Aim of the Study	Loss to Follow Up Less Than 5%	Prospective Calculation of the Study Size	Total
Ahlquist 2020 [17]	2	2	0	2	0	1	2	2	11
Allahabadi 2022 [18]	2	2	0	2	1	1	2	2	12
Allahabadi 2023 [19]	2	2	0	2	1	2	2	2	13
Arede 2019 [20]	2	2	0	2	0	2	1	2	11
Arnold 2019 [21]	2	0	0	2	1	2	1	2	10
Barfield 2019 [22]	2	1	0	2	1	2	2	2	12
Beese 2015 [23]	2	2	0	2	1	2	2	2	12
Bell 2016 [24]	2	1	0	2	1	2	1	2	11
Biese 2020a [25]	2	2	0	2	2	2	0	2	12
Biese 2020b [26]	2	0	0	2	2	2	0	2	10
Biese 2021 [27]	2	1	0	2	2	2	1	2	12
Biese 2022 [28]	2	1	0	1	1	2	1	2	10
Biese 2024 [29]	2	0	0	2	1	2	1	2	10
Bonnette 2023 [30]	2	2	0	2	1	2	2	2	13
Bridge 2013 [31]	2	1	0	2	1	2	1	2	11
Bush 2021 [32]	2	2	0	2	2	2	1	2	13
Butler 2024 [33]	2	1	0	2	1	2	1	2	11
Camp 2023 [34]	2	2	0	1	1	1	1	2	10
Chen 2022 [35]	2	0	0	2	0	2	1	2	9
Chou 2023 [36]	2	1	0	2	1	2	2	2	12
Confino 2019 [37]	2	1	0	1	0	2	2	2	10
Croci 2021 [38]	2	1	0	2	1	2	2	2	12
Dahab 2019 [39]	2	2	0	2	1	2	1	2	12
DiCesare 2019a [40]	2	1	0	2	0	2	1	2	10
DiCesare 2019b [41]	2	2	0	2	0	2	1	2	11
DiStefano 2018 [42]	2	2	0	1	2	2	1	2	12
Dobsacha 2023 [43]	2	0	0	2	1	2	0	2	9
Field 2019 [44]	2	1	0	2	1	2	0	2	10
Fransen 2012 [45]	2	2	0	2	1	2	2	2	13
Frome 2019 [46]	2	1	0	2	1	2	0	2	10
Gallant 2017 [47]	2	2	0	2	1	2	0	2	11
Garcia 2021a [48]	2	1	0	2	1	2	0	2	10
Garcia 2021b [49]	2	1	0	2	1	2	0	2	10
Ha 2023 [50]	1	2	0	2	2	2	1	2	12
Hall 2015 [51]	2	1	0	2	1	2	1	2	11
Heath 2021 [52]	1	2	0	2	1	2	1	2	11
Herman 2019 [53]	2	1	0	2	1	2	1	2	11
HuardPelletier 2022 [54]	2	2	0	2	0	2	1	2	11
HuardPelletier 2024 [55]	2	1	0	2	1	2	1	2	11
Iona 2022 [56]	2	0	0	2	0	2	0	2	8
Jayanthi 2015 [57]	2	2	0	2	1	2	1	2	12
Jayanthi 2020 [58]	2	2	0	1	1	2	0	2	10
Larson 2019 [59]	2	2	0	2	0	2	1	2	11
Lear 2024 [60]	2	2	0	2	0	2	0	2	10
Lenz 2024 [61]	2	2	0	1	2	1	1	2	11
Lima 2020 [62]	1	1	0	2	1	2	1	2	10
Li 2023 [63]	2	2	0	2	0	2	1	2	11
McDonald 2019 [64]	2	0	0	2	1	2	1	2	10
McGowan 2020 [65]	2	1	0	2	0	2	1	2	10
McGuine 2017 [66]	2	2	0	2	0	2	1	2	11
McKay 2023 [67]	2	2	0	2	0	2	1	2	11
Meisel 2022 [68]	2	2	0	2	0	2	0	2	10
Miller 2017 [69]	2	2	0	2	0	2	1	2	11
Moseid 2019 [70]	2	2	0	2	0	2	1	2	11
Murday 2024 [71]	2	2	0	2	0	2	1	2	11
Nagano 2023 [72]	2	2	0	2	0	2	1	2	11
Nguyen 2023 [73]	2	2	0	2	0	2	1	2	11
Okoruwa 2022 [74]	2	1	0	2	1	2	2	2	12
Pasulka 2017 [75]	2	2	0	2	1	2	1	2	13
Post 2017a [76]	2	2	0	2	0	2	1	2	11
Post 2017b [13]	2	2	0	2	1	2	1	2	12
Post 2020a [77]	2	1	0	2	0	2	1	2	10
Post 2020b [78]	2	2	0	2	1	2	1	2	12
Post 2021a [79]	2	2	0	2	1	2	1	2	12
Post 2021b [80]	2	2	0	2	1	2	1	2	12
Post 2021c [81]	2	2	0	2	1	2	1	2	12
Post 2024 [82]	2	2	0	2	1	2	1	2	12
Rauh 2020 [83]	2	2	0	2	1	1	1	2	11
Riehm 2023 [84]	2	1	0	2	1	2	1	2	11
Root 2019 [85]	2	2	0	2	1	2	2	2	13
Ross 2022 [86]	2	1	0	2	1	1	1	2	10
Rugg 2018 [87]	2	1	0	2	0	2	2	2	11
Rugg 2021 [88]	2	1	0	2	1	2	1	2	11
Sheppard 2020 [89]	2	2	0	2	0	2	1	2	11
Soderstrom 2023 [90]	0	2	0	2	1	2	0	2	9
Staub 2020 [91]	2	2	0	2	2	2	2	2	14
Steinl 2021 [10]	2	2	0	2	2	2	1	2	13
Stockbower 2022 [92]	2	2	0	2	2	2	1	2	13
Sugimoto 2019 [93]	2	2	0	2	2	2	1	2	13
Sweeney 2021 [94]	2	2	0	2	0	2	1	2	11
Swindell 2019 [11]	2	1	0	2	2	2	0	2	11
Valenzuela-Moss 2024 [95]	2	2	0	2	2	2	1	2	14
Vernick 2021 [96]	2	2	0	2	2	2	1	2	13
Watson 2019 [97]	2	2	0	2	2	2	1	2	13
Watson 2022 [98]	2	2	0	2	2	2	1	2	13
Whatman 2023 [99]	2	2	0	2	2	2	1	2	13
Whatman 2021 [100]	2	2	1	2	1	2	1	2	13
Wilhelm 2017 [101]	2	2	0	2	1	2	1	1	11
Wilkins 2023 [102]	2	2	0	2	1	2	1	1	11
Wilkins 2024 [103]	2	2	0	2	0	2	1	0	9
Xiao 2021 [104]	2	2	0	2	0	2	1	2	11
Zeller 2024 [105]	2	2	0	2	2	2	1	2	13
Zoellner 2022 [106]	2	2	0	2	1	2	2	2	13
